# Supplementary material for: Using the first nationwide survey on non-communicable disease risk factors and different definitions to evaluate the prevalence of metabolic syndrome in Burkina Faso
Source: PLoS One. 2021 Aug 5;16(8):e0255575. doi: 10.1371/journal.pone.0255575 (PMC8341491; doi:10.1371/journal.pone.0255575)
Supplement: Attachment — Submitted filename: Responses_to_Editor_PLONE_def2.docx [file pone.0255575.s001.docx]

Dear Editor,

Thank you for your request. We have addressed it in the manuscript by reformulating the Data availability section. We also added response in this letter (response is red in the text). We hope that this reformulation is satisfactory. We are still available if further information is needed.

Thank you very much.

Dr Kadari Cissé

=============================================

IMPORTANT: PLEASE DO NOT REPLY TO THIS EMAIL

If you are unable to complete any points that are requested in this email, please explain why in the "Enter Comments" tab of the online submission form prior to re-submitting your manuscript.  This will enable us to promptly assess your response and progress your manuscript to an Academic Editor at the earliest opportunity.

=============================================

PONE-D-20-38221R1

Using the first nationwide survey on non-communicable disease risk factors and different definitions to evaluate the prevalence of metabolic syndrome in Burkina Faso

Dr Kadari Cisse

Dear Dr Cisse,

Thank you for submitting your manuscript entitled "Using the first nationwide survey on non-communicable disease risk factors and different definitions to evaluate the prevalence of metabolic syndrome in Burkina Faso" to PLOS ONE.  Your manuscript files have been checked in-house but before we can proceed we need you to address the following issues:

1. We note that you have indicated that data from this study are available upon request. PLOS only allows data to be available upon request if there are legal or ethical restrictions on sharing data publicly. For more information on unacceptable data access restrictions, please see <http://journals.plos.org/plosone/s/data-availability#loc-unacceptable-data-access-restrictions>.

Dear Editor,

The STEPS survey data is owned by the Ministry of Health. The authors cannot share the dataset. However, interested researchers can access to the dataset by applying to ministry of health (contact: Tel: **(+226) 25 25 25 25; email:** [contact@sante.gov.bf](mailto:contact@sante.gov.bf)**) or by emailing directly the NCDs program coordinator Dr Paulin Somda:** [k_admos@yahoo.fr](mailto:k_admos@yahoo.fr).

Before we proceed with your manuscript, please address the following prompts:

a) If there are ethical or legal restrictions on sharing a de-identified data set, please explain them in detail (e.g., data contain potentially identifying or sensitive patient information, data are owned by a third-party organization, etc.) and who has imposed them (e.g., a Research Ethics Committee or Institutional Review Board, etc.). Please also provide contact information for a data access committee, ethics committee, or other institutional body to which data requests may be sent.

Dear Editor,

Thank you for this remark.

We have reformulated the Data availability section accordingly as follow:

**“The dataset of the STEPS survey that was used in this research is available at the Ministry of Health upon request since the data are owned by a third-party organization (ministry of health). The restriction was imposed by the Ministry of Health. Individuals interested can access to the STEPS survey dataset by emailing the ministry of health (****Tel: (+226) 25 25 25 25; email:** [**contact@sante.gov.bf**](mailto:contact@sante.gov.bf)**) or directly Dr Paulin Somda:** **[k_admos@yahoo.fr](mailto:k_admos@yahoo.fr) (NCDs program coordinator) or Zoma Torez :** [**torezo2000@yahoo.fr**](mailto:torezo2000@yahoo.fr) **(STEP survey data manager) ). All survey materials of STEPS survey are available on the WHO website (**[**https://extranet.who.int/ncdsmicrodata/index.php/catalog**](https://extranet.who.int/ncdsmicrodata/index.php/catalog)**). The authors confirm that they did not have any special access privileges that others would not have.”**

b) If there are no restrictions, please upload the minimal anonymized data set necessary to replicate your study findings to a stable, public repository and provide us with the relevant URLs, DOIs, or accession numbers. For a list of recommended repositories, please see

<https://journals.plos.org/plosone/s/recommended-repositories.> You also have the option of uploading the data as Supporting Information files, but we would recommend depositing data directly to a data repository if possible.

We will update your Data Availability statement on your behalf to reflect the information you provide.

Your manuscript has been returned to your account. Please log on to PLOS Editorial Manager at <https://www.editorialmanager.com/pone/> to access your manuscript.

Your manuscript can be found in the "Revisions Sent Back to the Author" link under the New Submissions menu. After you have made the changes requested above, please be sure to view and approve the revised PDF after rebuilding the PDF to complete the resubmission process.

Please note that these changes have been requested to comply with submission guidelines and your manuscript will *not* be sent to review until you have fully adhered to our requests.  Once your paper has been seen by an Editor we may return it to you for further information or amendments.

We ask that you address this request within 21 days. If you require additional time, please email the journal office. We are happy to grant extensions of up to one month past this due date. If we have not heard from you within 21 days, your manuscript will be withdrawn from Editorial Manager.

Thank you for submitting your work to PLOS ONE.

Kind regards,

Anna Mentsl

PLOS ONE

Thank you very much for the opportunity to make these changes.

__________________________________________________

In compliance with data protection regulations, you may request that we remove your personal registration details at any time.  (Use the following URL: <https://www.editorialmanager.com/pone/login.asp?a=r>). Please contact the publication office if you have any questions.
